# A Digital, Patient-Centred Carepath for Cardiac Surgical Care With Continuous Remote Patient Monitoring

**DOI:** 10.1093/icvts/ivag253

**Published:** 2026-09-11

**Authors:** Guylian Stevens, Jelle Fleerakkers, Jules François, Phillippe Ballaux, Koen Cathenis, Pascal Verdonck

**Affiliations:** Department of Chief Technology Officer, Maria Middelares VZW, 9000 Ghent, Belgium; Cardiac Surgery Department, AZ Maria Middelares, Buitenring Sint-Denijs, 9000 Ghent, Belgium; Cardiac Surgery Department, AZ Maria Middelares, Buitenring Sint-Denijs, 9000 Ghent, Belgium; Cardiac Surgery Department, AZ Maria Middelares, Buitenring Sint-Denijs, 9000 Ghent, Belgium; Cardiac Surgery Department, AZ Maria Middelares, Buitenring Sint-Denijs, 9000 Ghent, Belgium; Department of Electronics and Information Systems—IBiTech, Ghent University, 9000 Gent, Belgium

**Keywords:** digital health, patient remote monitoring, cardiac surgery, enhanced recovery

## Abstract

Postoperative complications and readmissions remain common after cardiac surgery, and intermittent manual vital-sign checks leave patients unmonitored for much of the recovery period. We implemented a phygital care pathway combining enhanced recovery after surgery (ERAS) protocols, and 21-day continuous remote monitoring (heart rate, respiratory rate, temperature, blood pressure, and oxygen saturation) with daily review and escalation to a cardiac surgeon when indicated. Quality of recovery (QoR-15) was assessed on preoperative days −1 and postoperative days 3 and 7, and patient-reported experience measurement (PREM) on day 30. Outcomes in 221 monitored patients (May-November 2025) were compared, retrospectively, with a historical, non-monitored cohort of 206 patients. In the monitored patients, a good-to-excellent QoR increase from 25% to 56% between days 3 and 7 was noticed. Seventy-five patients (34%) required 98 interventions, most commonly primary-care referral or medication adjustment. Thirty-day readmission (12.7% vs 12.1%, Fisher *P* = .88) and mortality (0.45% vs 1.4%, Fisher *P* = .36) did not differ significantly from the control cohort; the monitored group was significantly younger (66.7 vs 70.1 years, *P* = .0004), reflecting probable selection bias from smartphone and digital-literacy eligibility criteria. Patient-reported experience measurement scores were high (>91%). This pilot demonstrates the feasibility of a digital, patient-centred cardiac surgical pathway; readmission and mortality findings are hypothesis-generating and require prospective confirmation.

## INTRODUCTION

Despite advances in minimally invasive techniques and perioperative care, postoperative complications, prolonged hospital stays, and readmissions remain common after cardiac surgery.^1^ Global health-workforce shortages are projected to reach 11 million by 2030,^2^^,^^3^ putting pressure on hospital capacities, leading to earlier discharge and the need for closer follow-up during the first period of recovery at home. Initiatives with manual vital-sign measurements exist, but such intermittent, effort-dependent assessments are prone to omission, and spot-check measurements of parameters such as respiratory rate are frequently inaccurate.^3^^,^^4^

Remote patient monitoring (RPM) enables continuous assessment of physiological parameters and has shown potential to support proactive, individualized postoperative care.^5^^,^^6^ Remote patient monitoring interventions demonstrate positive outcomes in patient safety and adherence, improve patients’ mobility and functional statuses, and has the possibility to decrease the risks of hospital admission/readmission, length of stay, number of outpatient visits, and non-hospitalization costs.^7^

This brief communication describes the implementation of a digital, patient-centred care pathway integrating enhanced recovery after surgery (ERAS) protocols, and continuous remote monitoring. The primary objective was to assess the feasibility of this pathway in routine practice; secondary, descriptive objectives were postoperative recovery, patient-reported experience, and 30-day readmission and mortality relative to a historical cohort.

## METHODS

### Ethical approval declarations

This retrospective observational analysis of prospectively collected data compared with a historical control cohort collected remote-monitoring data from a digital care pathway implemented at Maria Middelares (Ghent, Belgium), compared with a historical, non-monitored control cohort. The study was approved by the Maria Middelares ethics committee (RWE-2025-035); written informed consent was obtained from all monitored patients, and the study followed the Declaration of Helsinki.

### The implemented pathway

The pathway (**Figure 1**) combined 3 elements: (1) ERAS protocols (multimodal analgesia, atrial fibrillation prevention, early mobilization), delivered via a multidisciplinary preoperative consultation (“Heart Carousel”), (2) systematic quality of recovery (QoR)-15 assessment (postoperative days 3 and 7) and patient-reported experience measurement (PREM) assessment (day 30), and (3) 21 days of continuous postoperative monitoring (heart rate, respiratory rate, temperature, non-invasive blood pressure, SpO2, spot-check ECG, and atrial fibrillation detection) via a wearable device (Corsano), reviewed daily by a dedicated nursing team through a standardized protocol with escalation to a cardiac surgeon when indicated, alongside weekly structured telephone check-ins.

**Figure 1.**
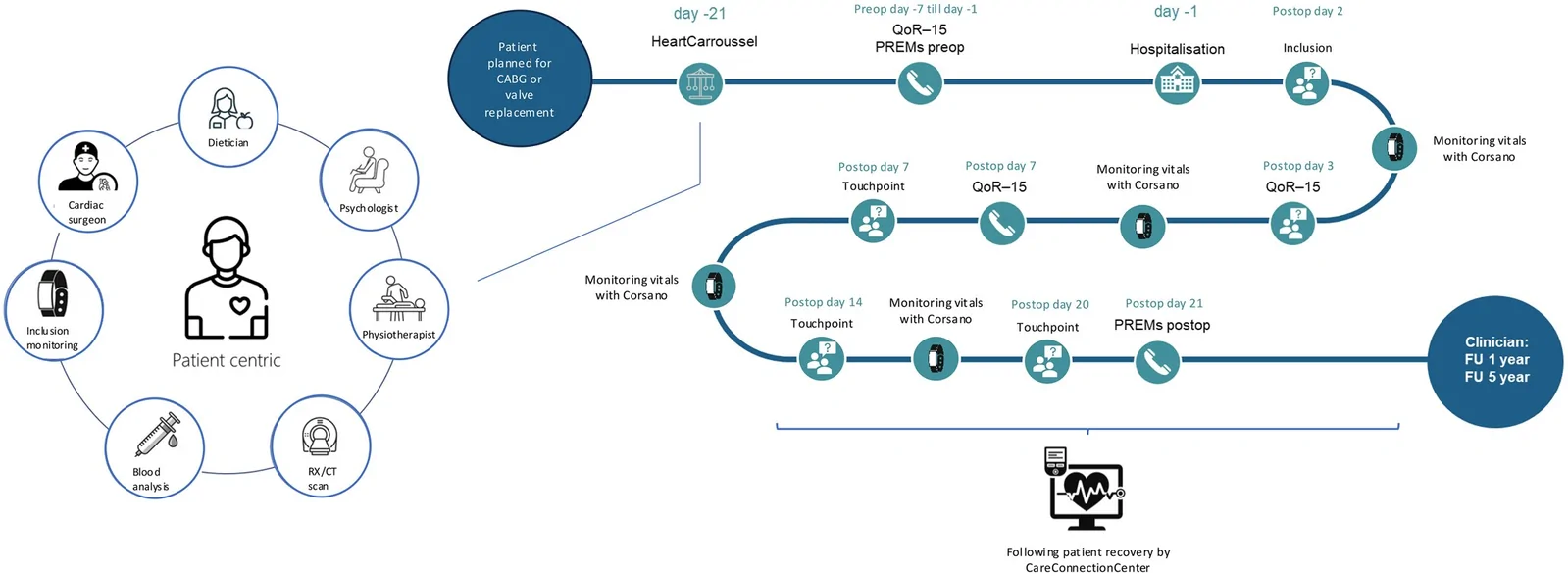
Process flow of the digital, value-based care pathway in cardiac surgery including preoperative screening, Quality of Recovery and Patient reported experience measurement questionnairs and 21d of continuous vital sign monitoring

All patients undergoing cardiac surgery were offered enrolment, contingent on informed consent and possession of a compatible smartphone (iOS ≥15 or Android ≥8.0, Bluetooth 5.0 LE). Between May and November 2025, 221 patients were enrolled; 53 additional patients were excluded, mainly for lack of a compatible smartphone or limited digital literacy. All patients were analysed on an intention-to-treat basis, including 13 patients (6%) who discontinued monitoring before day 21 due to anxiety, stress, or discomfort. A historical, consecutive cohort (under the same clinical policy) of 206 non-monitored patients from an earlier 3-month (January 2026 until April 2026) period served as the control. Outcomes for both cohorts were obtained by manual electronic health record (EHR) review.

All patient monitoring data were summarized in a concise report, including the daily maximum, minimum, and mean values for each of the 5 measured vital signs. The resulting reports were made available through the Flemish care collaboration platform, CoZo, enabling general practitioners and cardiologists involved in the patient’s care to access and review the monitoring data.

### Statistical analysis

Categorical outcomes (30-day readmission, mortality) were compared between cohorts using Fisher’s exact test; the continuous baseline variable age was compared using an independent-samples *t*-test. A 2-sided *P* < .05 was considered statistically significant. Analyses were descriptive/exploratory given the retrospective, non-randomized design and were not powered for a definitive comparative-effectiveness conclusion.

## RESULTS

Patient characteristics and outcomes are summarized in **Table 1**. The monitored cohort was significantly younger than the historical control (66.7 ± 10.4 vs 70.1 ± 9.2 years, *P* = .0004); sex distribution and procedure mix were similar between groups.

**Table 1.** Patient Characteristics and Key Outcomes

	Monitored (*n* = 221)	Non-monitored/historical control (*n* = 206)	*P*-value
Age, years (mean ± SD)	66.7 ± 10.4	70.1 ± 9.2	.0004
Male sex, *n* (%)	162 (77%)	157 (76%)	—
Euroscore II			
CABG, *n* (%)	119 (56%)	125 (61%)	—
Isolated aortic valve, *n* (%)	35 (17%)	30 (15%)	—
Isolated mitral valve, *n* (%)	30 (14%)	25 (12%)	—
Combined procedure, *n* (%)	17 (8%)	14 (7%)	—
Aortic surgery, *n* (%)	6 (3%)	8 (4%)	—
30-day readmission, *n* (%)	28 (12.7%)	25 (12.1%)	.88
30-day mortality, *n* (%)	1 (0.45%)	3 (1.4%)	.36
Good–excellent QoR-15, day −1 → day 3	66% → 25%	—	—
Good–excellent QoR-15, day 3 → day 7	25% → 56%	—	—
PREM: care coordination	95.4%	—	—
PREM: professional expertise	97.7%	—	—
PREM: felt adequately informed	91.7%	—	—

*P*-values: Fisher’s exact test (categorical)/independent-samples *t*-test (age); “—” = not formally tested or not collected for this cohort.

Good-to-excellent (QoR-15) recovery first decreased from 66% preoperative to 25% on postoperative day 3. After surgery, it increased from 25% on postoperative day 3% to 56% on day 7, while poor recovery decreased from 12.4% to 6%. Day-30 PREM scores indicated high satisfaction with care coordination (95.4%), professional expertise (97.7%), and feeling adequately informed (91.7%); no PREM data were collected for the historical cohort.

Seventy-five of 221 monitored patients (34%) required at least 1 intervention by the follow-up team; 98 interventions were recorded overall (1.3 per intervened patient), comprising 5 hospital-readmission advisories, 10 emergency-department referrals, 44 primary-care/cardiologist referrals, 4 direct cardiac-surgeon consultations, and 35 medication or therapy adjustments.

Thirty-day readmission occurred in 28/221 (12.7%) monitored patients versus 25/206 (12.1%) in the historical control cohort; this difference was not statistically significant (Fisher’s exact *P* = .88). Thirty-day mortality occurred in 1/221 (0.45%) monitored patients versus 3/206 (1.4%) controls (*P* = .36). Given the small event numbers, the significantly younger age of the monitored cohort, and the unadjusted, retrospective comparison, these readmission and mortality findings should be interpreted as hypothesis-generating rather than as evidence of clinical benefit.

Illustrative use-cases: in a first patient, on postoperative day 12, continuous monitoring flagged new-onset tachyarrhythmia (heart rate up to 150 bpm) in an otherwise stable patient, prompting same-day electrocardiographic evaluation and emergency-department referral; the patient was diagnosed with a pericardial effusion, treated conservatively, and resumed structured remote follow-up after discharge without further deterioration. In a second patient, remote monitoring detected nocturnal low-grade fever 11 days after discharge, leading to timely diagnosis and treatment of a wound infection.

## LIMITATIONS AND STRENGTHS

This pilot has important limitations. The retrospective, non-randomized, single-centre design with a historical control introduces risk of temporal confounding and unmeasured bias; a randomized design was not undertaken because the digital, patient-centred pathway had already been adopted as standard of care. The smartphone/digital-literacy eligibility requirement (53 patients excluded) likely selected a younger, healthier cohort, consistent with the observed age imbalance, and may have influenced the outcome comparisons above. Documentation of patient-care team interactions relied on manual EHR review without a standardized framework, which may affect completeness; structured Tiro.Health forms are being implemented to address this. Findings from a single Belgian centre may not generalize to healthcare systems with different discharge practices, digital literacy, or follow-up infrastructure.

Strengths include real-world, consecutive enrolment reflecting routine clinical implementation rather than an idealized trial protocol, which supports the feasibility conclusions drawn here, though not claims of comparative effectiveness.

## CONCLUSION

This pilot study demonstrates the feasibility of implementing a phygital, patient-centred care pathway integrating minimally invasive cardiac surgery, ERAS, and continuous remote monitoring in routine practice. The pathway was associated with within-group improvement in QoR between postoperative days 3 and 7, high patient-reported experience, and 30-day readmission and mortality rates that were numerically similar to a historical, non-monitored cohort, without reaching statistical significance. These findings are hypothesis-generating and should be confirmed in prospective, adequately powered, ideally multicentre studies with structured baseline and cost data before conclusions about clinical or economic benefit can be drawn.

## Data Availability

The data underlying this article will be shared on reasonable request to the corresponding author.

## Abbreviations

EHR: electronic health record
ERAS: enhanced recovery after surgery
PREM: patient-reported experience measurement
PROM: patient-reported outcome measurement
QoR: quality of recovery
RPM: remote patient monitoring
